# Congenital cervical fusion in Klippel-Feil syndrome with omovertebral bone: a rare clinical image

**DOI:** 10.11604/pamj.2024.49.96.45616

**Published:** 2024-11-27

**Authors:** Devyansh Nimodia, Anand Hatgaonkar

**Affiliations:** 1 Department of Radiodiagnosis, Datta Meghe Institute of Medical Sciences, Sawangi, Wardha, Maharashtra, India,; 2 Department of Radiodiagnosis, Datta Meghe Medical College, Nagpur, Maharashtra, India

**Keywords:** Klippel-Feil syndrome, cervical spine injuries, congenital fusion

## Image in medicine

A 31-year-old male presented to the emergency department after a road traffic accident. Computed tomography scan imaging revealed that the patient had congenital fusion at two levels of the cervical spine, C2-C3 and C3-C4 and C5, with an osseous overgrowth at the level of C3-C4 involving the left lamina with extension into the paravertebral region suggestive of omovertebral bone. Additionally, the patient had a Sprengel deformity, characterized by a higher position of the scapulae. The patient's accident resulted in spinal cord compression and cord contusion extending from the C3-C4 intervertebral disc superiorly to the level of C5-C6 inferiorly and superiorly to the C3 mid-vertebral level. Associated syrinx was also noted. The patient was diagnosed with Klippel-Feil syndrome type II due to the numerous structural abnormalities and lack of lower thoracic or lumbar fusion. C2-C3 is the level of congenital fusion that is most typical. Klippel-Feil syndrome is a rare congenital condition that affects the development of the cervical spine and is characterized by the fusion of two or more cervical vertebrae. Patients with Klippel-Feil syndrome may present with a triad of symptoms, including a short neck, restricted neck or head movement, and a receding hairline in the back. Management of Klippel-Feil syndrome is typically supportive, and lifestyle modifications such as avoiding contact sports, using neck braces, and traction can provide symptomatic relief. However, if conservative management fails, patients with adverse sequelae such as pain or progressive instability may benefit from surgical decompression, with or without fusion. In conclusion, Klippel-Feil syndrome is a rare condition that requires a comprehensive approach to management. This case highlights the importance of considering Klippel-Feil syndrome in patients with cervical spine injuries and the need for close monitoring and timely intervention to prevent complications and improve patient outcomes.

**Figure 1:**
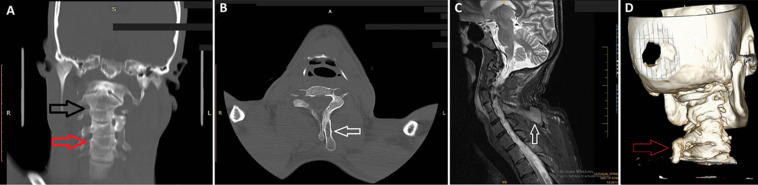
A) computed tomography scan bone window coronal image showing congenital fusion at two levels of the cervical spine, C2-C3 (black arrow) and C3-C4 and C5 (red arrow); B) computed tomography axial bone window image; C) sagittal STIR image (open arrow) showing an osseous overgrowth at the level of C3-C4 involving the left lamina with extension into the paravertebral region suggestive of omovertebral bone and two spinous process; D) computed tomography reformatted image showing omovertebral bone

